# Recent Advances in the Development of Antibiotics-Coated Gold Nanoparticles to Combat Antimicrobial Resistance

**DOI:** 10.3390/antibiotics13020124

**Published:** 2024-01-26

**Authors:** Partha Pratim Sarma, Akhilesh Rai, Pranjal K. Baruah

**Affiliations:** 1 Department of Applied Sciences, GUIST, Gauhati University, Guwahati 781014, Assam, India; 2 CNC—Center for Neuroscience and Cell Biology and Center for Innovative Biomedicine and Biotechnology, University of Coimbra, 000-447 Coimbra, Portugal

**Keywords:** antimicrobial resistance, antibiotics, gold nanoparticles, antimicrobial properties, drug delivery

## Abstract

Antimicrobial resistance (AMR) has become an alarming threat to the successful treatment of rapidly growing bacterial infections due to the abuse and misuse of antibiotics. Traditional antibiotics bear many limitations, including restricted bioavailability, inadequate penetration and the emergence of antimicrobial-resistant microorganisms. Recent advances in nanotechnology for the introduction of nanoparticles with fascinating physicochemical characteristics have been predicted as an innovative means of defence against antimicrobial-resistant diseases. The use of nanoparticles provides several benefits, including improved tissue targeting, better solubility, improved stability, enhanced epithelial permeability and causes minimal side effects. However, except for gold nanoparticles (AuNPs), the biological safety of the majority of metal nanoparticles remains a serious problem. AuNPs appear to be promising for drug delivery and medicinal applications because of their minimal toxicity, biocompatibility, functional flexibility, chemical stability and versatile biological activities, such as their antiviral, antifungal, anti-inflammatory and antimicrobial properties. Hence, we are focusing on the gold nanoparticles possessing antimicrobial activity in this article. This review will cover recent strategies in the preparation of gold nanoparticles, with special emphasis placed on antibiotics-coated AuNPs with enhanced antimicrobial properties and how they fight against disease-causing bacteria and eradicate biofilms, along with their activities and physicochemical properties.

## 1. Introduction

The tremendous increase in antibiotic resistance among pathogenic microorganisms is a major hazard to human wellness. Antibiotics can be regarded as the organic compounds that suppress the growth of or kill bacterial cells, thereby preventing infections caused by pathogenic bacteria [1,2]. Antibiotics are considered as vital in practically every major therapeutic field, including serious surgeries, notably the transplantation of organs, premature newborn treatment, and chemotherapy medication in cancer patients, which could not be achieved without adequately avoiding and treating bacterial infections [3,4]. The most frequently used antibacterial medications are β-lactam antibiotics; yet, because of growing bacterial resistance, several drugs of this class have loosened their clinical effectiveness [5]. Furthermore, using antibiotics at higher doses during treatment promotes the emergence of multidrug resistance (MDR), rendering antibiotic therapy ineffective against pathogenic bacteria [6]. However, another important factor that contributes to the development of MDR in bacteria is the uses of sub-lethal doses of antibiotics, wherein bacteria gain resistance to drugs without being killed. Incorrect and excessive antibiotic administration, insufficient diagnosis, and advanced resistance mechanisms accrued by microorganisms can all lead to collapses of therapeutic efficiency [7,8]. Biofilm-associated illnesses which have become tolerant to current antibiotic treatments leading to scarcity of efficient therapeutic alternatives have become a serious matter of concern nowadays. Besides these, the other contributing factors for increased AMR include the widespread use of antibiotics in the field of agriculture as well as in dairy and poultry industries, a lack of novel medications, massive regulatory hurdles, incorrect prescriptions, etc. [9,10]. The inadequate effectiveness of traditional antibiotics, whether due to extended-spectrum β-lactamases (ESBLs) or through other resistance pathways, necessitates the innovation of novel therapeutic remedies with enhanced activities. Furthermore, combating infections using as low a dose as possible might serve as the most efficient approach to fight against diseases caused by MDR bacteria [7]. While developing new antimicrobial compounds to fight drug-resistant bacteria is extremely difficult, it is also extremely crucial and necessary in the interim. Chemical alteration in antibiotic structures to combat antimicrobial resistance as well as the synthesis of novel antibiotics with high efficiency is arduous and is typically not economically viable. To render antibiotic therapy more efficient and cost-effective, the antibiotic dose must be reduced while the stability must be enhanced [11].

Recent advances in medical research introduced novel antibiotics and advanced clinical therapies that can effectively tackle pathogenic microorganisms [12]. The application of nanomaterials in the domain of antibiotic drug delivery offers the potential for significant improvements in the clinical effectiveness of antibacterial treatment. In comparison to traditional medications, nanoformulations for the administration of antibiotics along with infectious site targeting provide several advantages, which comprise enhanced tissue targeting, prolonged antibiotic half-life, raised solubility, gained greater stability, increased permeability to epithelial cells, and limited side effects [13,14]. Recently, nanoparticles (NPs) (having a particle size between 1 and 100 nm) have come into the limelight as a revolutionary approach for treating deadly diseases caused by bacteria. Moreover, metallic NPs functionalised with antibiotics offer promising nanoplatforms for fighting against bacterial resistance.

The use of nanomaterials in medicine has also historical importance in India. Bhasmas, which have been utilised as Ayurvedic medicines in India to cure a variety of diseases including cancer, were discovered to contain NPs of alloys, sulphides, metals and metal oxides [1,15]. Nanomaterials possess promising potential to be used in the field of drug delivery, medical imaging and disease diagnosis, considering their high surface area as well as their small size effect. Among the diverse range of nanomaterials, NPs like AgNPs, AuNPs, ZnONPs and CuONPs have been receiving specific attention because of their easy and simple synthesis procedure, higher biocompatibility and versatile physicochemical properties that can be easily altered by stimulating parameters like temperature, pH, light and reaction time [16,17,18]. NPs exhibit the tremendous potential to serve as drug carriers and can be easily functionalised by incorporating bioactive compounds, drug molecules, antibiotics, polymers, antibacterial peptides, etc. for the enhancement of antimicrobial activity to combat AMR [19,20,21]. Polymer-functionalised NPs have offered immense promises in the field of novel drug carrier and delivery systems owing to their great potential for protecting as well as improving the bioavailability and release rate of encapsulated drug molecules thereby contributing to the reduction in toxic impacts [21]. Because of their flexible physical characteristics, NPs serve as a versatile system for medicinal applications. The small size of the nanomaterials imparts a high surface-to-volume ratio, which promotes the binding of several antibacterial agents in order to produce multivalent nanomaterials against pathogenic bacteria [7]. More importantly, many NPs demonstrate inherent antimicrobial characteristics by inhibiting biofilm formation, activating reactive oxygen production, and interfering with bacterial cell membranes as well as with proteins and DNA within the bacterial cell [16]. Several metal NPs, such as Ag, Au, Cu, Zn, Ce, Mg, Pd, Ti, etc., and metal oxide NPs, such as ZnO, CuO, NiO, Al_2_O_3_ TiO and Fe_3_O_2_, as well as their functionalisation through conjugation with other compounds, have been reported to demonstrate antibacterial properties against various pathogenic bacteria [16,22,23,24,25]. Compared to the pure AuNPs, the conjugated or functionalised AuNPs demonstrate better antimicrobial properties [19,20,21]. Since NPs do not exhibit a specific mechanism of action unlike antibiotics, these are especially advantageous in combating bacterial resistance [1]. Recent studies revealed that AuNPs do not impart any toxic effects in human cells; hence, AuNPs have captured the interest of modern biomedical researchers [26]. AgNPs are also used in medicinal therapies. Although AgNPs are easy to synthesise, cost effective and possess inherent antimicrobial property, their cytotoxicity due to the release as well as aggregation of Ag ions in the body from AgNPs has restricted their utilisation in nanomedicine. However, the higher biocompatibility ease of synthesis and inertness of the AuNPs make them suitable for use in medicinal therapies. High cost is the major disadvantage associated with AuNPs [27,28]. The higher biocompatibility, nontoxicity, high rates of absorption and powerful light scattering allow them to be used in versatile fields, such as electronics, sensors, catalysis, drug delivery, drug carriers and other biomedical applications, including delivery of genes, molecular imaging, targeted drug administration, plasmonic bio-sensing, tissue engineering, colorimetric sensing, cancer treatment, diagnostics and photo-induced therapy [27,28,29]. Recently, AuNPs were employed to administer numerous antibiotics belonging to different classes that included carbapenems, polymyxins, tetracycline, cephalosporin, glycopeptide, cephalosporin, aminoglycoside, penicillin and cephalosporin [1,30,31]. However, numerous limitations are associated with modern as well as conventional antibiotic medication therapy, including limited bioavailability, the emergence of antibiotic-resistant bacteria, poor penetration power, insufficient drug concentrations at specific infection sites, adverse side effects, a higher frequency of administration, and poor patient compliance. Therefore, AuNP-conjugated drug delivery systems provide a superior and revolutionary approach to eradicating the disadvantages associated with the use of antimicrobial drug therapy, because of their large surface-to-volume ratio, photostability, ability to target biofilms, potential for organ as well as cellular targeting, strong interaction and their penetration through the bacterial cell wall [3,13]. The conjugation of antibiotic drug molecules with AuNPs significantly enhances their antimicrobial properties. The conjugation of antibiotic drugs with AuNPs leads to the enhanced bioavailability and biocompatibility of the drugs. This can also enhance the interaction as well as penetration power of the drug molecules though the bacterial cell wall. The electrostatic attraction between the negatively charged bacterial cell wall and the cationic behaviour of the antibiotic-loaded AuNPs enhances the interaction as well as penetrating power of the drug molecules through the bacterial cell wall. This facilitates the release of the antibiotic drug molecules within the bacterial cell and interacts with the bacterial cellular matrix causing the death of bacteria [5,6]. Indeed, AuNPs are effective for the transformation of various ineffective antibiotics into Au nanoformulations with strong antimicrobial properties [32]. However, the efficient incorporation of a specific antibiotic drug molecule on the surface of the AuNPs requires a variety of strategies, including physical absorption, electrostatic relations, coupling processes and Au-S and Au-N linkages [1]. Different physical, chemical and biological methods, including green synthesis, for the fabrication of AuNPs have been reported. Among these, chemical colloidal synthesis, which involves the use of a metal precursor as well as a reducing and a stabilising agent, is one of the most frequently employed methods for the synthesis of AuNPs [1,7].

**Figure 1:**
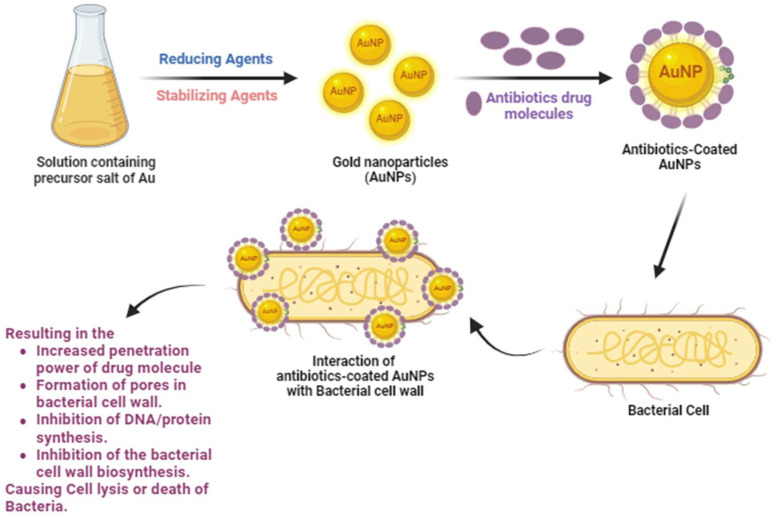
Synthesis of antibiotic-coated AuNPs and their interaction with the bacterial cell to combat AMR.

Thus, the review covers cutting-edge research on the effectiveness of AuNPs as a strategy for combating antimicrobial resistance. Special emphasis has been put on the development of bioactive and antibiotic-coated AuNPs exhibiting enhanced antimicrobial attributes to combat pathogenic bacteria and biofilms, as well as their activities and physicochemical features. A schematic representation of the synthesis of antibiotic-coated AuNPs and their interaction with bacterial cells to combat AMR is demonstrated in Figure 1.

## 2. AMR—A Global Threat

The World Health Organization (WHO) has recognised AMR as one of the leading global hazards to human health and development. It falls among the top ten public health threats worldwide [33]. The escalating growth and dissemination of antibiotic-resistant organisms and the inadequate efficacy of antimicrobial drugs make the treatment of antimicrobial infection challenging, leading to elevated fatality rates and huge monetary expenses [34,35]. With the current scientific revolutions, antibiotics have become a wonderful gift to the livestock and human healthcare professions to aid in the cure of bacterial infections as well as other disorders. Before the 1950s, antibiotics were frequently employed in both human health and animal husbandry due to their low cost and few side effects [13]. However, the serious problem of drug resistance has arisen as a result of the decades-long widespread use of antibiotics. The discovery of β-lactam antibiotics provided a temporary solution to the problem, but disappointingly, this did not endure for long with the first instance of methicillin-resistant *Staphylococcus aureus* (MRSA) identified in the UK in 1961 [27,36]. The ability of a specific microbe to avoid the pharmacological mechanism of action linked to antibacterial medications and then go on living is known as antibacterial resistance. The growing number of drug-resistant microorganisms has been strongly attributed to the inappropriate application of broad-spectrum antibiotics. *Klebsiella pneumoniae* is a multi-drug resistant (MDR) bacteria that causes several types of hospital-acquired illnesses and epidemics, including septicaemia, urinary tract infections and pneumonia [14]. It has developed resistance against carbapenem and tigecycline, which is the most effective as well as a last-resort antibiotic for treating *K. pneumoniae*. Due to the secretion of the β lactam enzymes like carbapenemases and extended-spectrum β-lactamases, *K. pneumoniae* is capable of hydrolysing a wide range of broad-spectrum β-lactam antibiotics [17,37]. *Salmonella* spp. responsible for diseases like bacteremia, gastroenteritis, paratyphoid and typhoid have become fluoroquinolone-resistant [38]. Infections like pneumonia, tuberculosis and gonorrhoea become challenging to cure since medications are becoming less effective towards these diseases because of their increased resistance of bacteria. Moreover, ESBLs have now developed resistance to several antibiotic classes, including aminoglycosides, tetracyclines, cotrimoxazole, quinolones and trimethoprim, further limiting therapeutic choices for clinicians [7,39]. Multidrug resistance has emerged as a global threat and is accelerating rapidly. Nowadays, MDR has become more prevalent in both pathogenic as well as non-pathogenic bacterial strains and is primarily attributed to gene acquisition and/or alteration in the target genes of antibiotics [9]. Besides these, the antibiotic resistance of bacteria was regarded as one of the major economic burdens for the world [9]. Lack of access to conventional antibiotics, however, will result in costlier medication, extended hospital exposure, prolonged treatment period, higher treatment expenses due to the need for intensive care and, ultimately, life-threatening harm [40]. The widespread usage of antibiotics during the COVID-19 pandemic has fuelled the issue of antimicrobial resistance. Antibiotics, antiviral and antipyretic medications that have been used erratically to treat COVID-19 have led to the emergence of AMR [41]. Some pathogenic bacteria that have developed resistance against multiple drugs and their harmful effects on humans are represented in Table 1.

## 3. Bacterial Resistance Mechanisms

**Figure 2:**
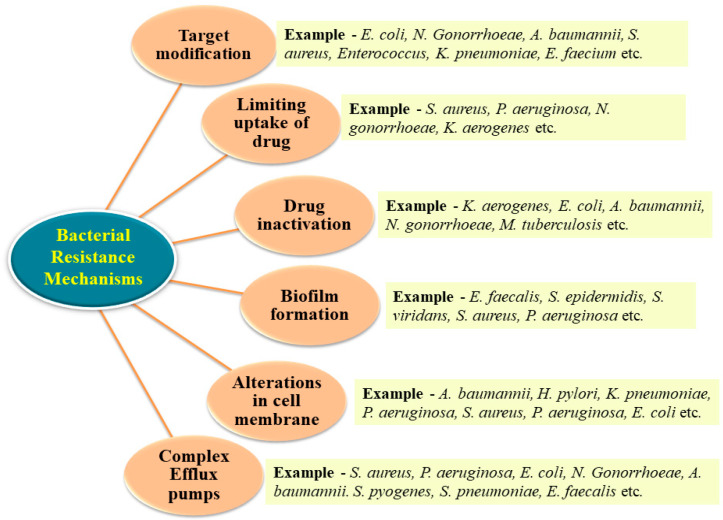
Bacterial approaches towards antibacterial resistance.

There are four primary mechanisms including the inactivation of drug molecules, target modification, limiting drug uptake and the development of efflux pumps through which bacteria can develop resistance towards antibiotics [9,51]. Some strategies adopted by bacteria to develop resistance against antibiotics are represented in Figure 2.

### 3.1. Inactivation of the Drug

In severe cases, bacteria acquire resistance through their direct interaction with the antibiotics via hydrolysis or the transfer of a chemical moiety including adenyl, acetyl and phosphoryl groups [51]. A wide range of Gram-positive and Gram-negative bacteria develop resistance to certain antibiotics through the secretion of enzymes that have the ability to bring out chemical modifications to the antibacterial compound. Among these, β-lactamases are the most prominent, which can demolish the drug molecule by hydrolysing the amide linkage of the β-lactam ring, rendering it ineffective. The most common biochemical reactions catalysed by modifying enzymes include acetylation, phosphorylation and adenylation [51,52].

### 3.2. Target Modification

Bacteria can modify the target of the antibiotic through protection, mutation and posttranslational modification [27]. Point mutations within the gene generating the target site, enzymatic transformations of the target and evading the original site are the primary components of target site alteration. Evading the original site, bacteria create new targets that resemble the original but are not exact replicas of it. As a result, antibiotics will be unable to inhibit the metabolic processes accomplished by newly developed equivalent targets [51,52].

### 3.3. Limiting Drug Uptake

The primary surface membrane component found in practically all Gram-negative bacteria is lipopolysaccharide (LPS), which is crucial to the outer membrane’s structure as well as its functionality [51,53]. The presence of this LPS layer provides a barrier to a large number of antibacterial agents. However, for Gram-positive bacteria, the outer membrane is absent. The presence of the outer membrane in the Gram-negative bacteria is the primary reason for the development of resistance towards a wide range of antibiotics. Modifications in this outer membrane, such as the mutation of porins or the alteration of hydrophobic properties, can impart resistance [51,53]. Hence, bacterial infections caused by Gram-negative bacteria are difficult to cure compared to Gram-positive bacterial infections. In Gram-negative bacteria, drug molecules penetrate the cell through porin channels. Bacteria can impede drug uptake by reducing the porin channels or by mutations that lead to alterations in the selectivity of the porin.

### 3.4. Development of Efflux Pump

Genes producing efflux pumps are found chromosomally in bacteria. Transmembrane proteins called efflux pumps enable bacteria to exchange or evacuate antibiotic compounds from their cells, which helps the bacteria to survive [51,54]. These are the bacteria’s self-defence mechanisms. Based on structure as well as energy sources, there are five major families of efflux pumps, which include the ATP binding cassette family, small multidrug resistance family, resistance–nodulation–cell division family, multidrug and toxic compound extrusion family and major facilitator superfamily [51].

## 4. Synthesis of Antibiotic-Coated AuNPs

A variety of physical, chemical or green synthesis techniques could be employed for the synthesis of AuNPs. The most frequently employed physical methods for the fabrication of AuNPs are laser ablation and evaporation–condensation [55]. The fundamental steps in the chemical synthesis method of AuNPs involve the formation of Au^0^ through the reduction of Au^+^ ions by the action of a reducing agent followed by stabilisation via capping agents [7]. During the chemical synthesis procedures, different chemicals were used as reducing and capping agents. The synthesis of AuNPs from vitamins, enzymes, microbes and plant extracts using green solvents is the prime focus of green synthesis methods. The green synthesized, antibiotic-loaded nanomaterials serve as a fascinating and significantly effective alternative for the treatment of disease caused by the microbes as they reduce or avoid the use of any hazardous compounds, thereby reducing side effects [25,56]. AuNPs may exhibit various colours depending on their shape, size, aggregation level and surrounding environment because of the surface plasmon resonance. A variation of colour from light pink to dark purple can appear owing to the localised surface plasmon resonance (SPR), which is further associated with the size and shape of the NPs [7,57]. AuNPs exhibit a SPR band between 500 and 550 nm [57]. However, the facile surface functionalisation of AuNPs makes it easier to conjugate versatile bioactive compounds or ligands with AuNPs which significantly enhances the functionality of the NPs. The incorporation of antibiotic drug molecules on the surface of AuNPs is a recent innovation and is reported to be highly efficient in combating bacterial resistance toward traditional antibiotics. In most of the studies, the antibiotic-coated AuNPs were fabricated using chemical methods. Antibiotics are able to adhere to the surface of AuNPs by means of their different functional groups, including amino, carboxylic, hydroxyl and thiols, resulting in the surface modification of AuNPs leading to the enhancement in the antibacterial activity of the system [58]. Temperature, reaction time and pH are the imminent factors that significantly affect the properties of the AuNPs. Recently, in 2023, Halawani et al. evaluated the effect of the concentration of precursor salt, temperature, reaction time and pH on the synthesis of amoxicillin-conjugated AuNPs [59]. They have reported that with an increase in the concentration of the precursor salt from 1 mM to 5 mM, the absorption spectra become more intense, showing a blue shift and revealing the decreased size of the synthesized AuNPs. Further increasing the concentration beyond 5 mM leads to aggregation and precipitation of the NPs indicating a decrease in stability. Time also possesses a crucial role in the synthesis of NPs. An increase in the absorption peak was reported up to 1 h; however, further increasing the reaction time showed a decrease in the intensity of the absorption spectra. They have reported that the ideal pH for the synthesis of AuNPs is 6.

## 5. Antibiotic-Coated AuNPs to Combat AMR

The emergence of bacterial resistance to conventional antibiotics is a worldwide concern for human health. The widespread usage of antibiotics among medical professionals and throughout the community is the driving force for the development of resistance to conventional antibiotics for a wide range of bacteria. This has disputed the clinical efficacy of certain present antimicrobial medicines and highlighted the demand for relatively more effective antibiotics. AuNPs, with these promising features, offer a diverse platform for nanoscale biological assemblies comprising antimicrobial agents, antibodies, nucleotide proteins and DNA [1]. Recently, the synergistic effects of AuNP-based formulation have attracted researchers. Formulations of antibiotic-coated AuNPs are undergoing clinical trials and have drawn significant attention. The activity of some recently developed antibiotic-coated AuNPs to combat AMR are represented briefly in Table 2.

Ali et al. prepared cefixime-stabilised AuNPs through an easy, simple as well as single-step procedure and evaluated the antibacterial activity against *S. aureus* bacteria [5]. *S. aureus* belongs to the groups of human pathogens that can trigger a variety of infections, including food poisoning as well as respiratory and skin infections. Because *S. aureus* has the ability to break down β-lactam antibiotics using lactamase, it could develop resistance to these drugs. Cefixime belongs to the class of drugs designated as cephalosporin antibiotics. A potent β-lactam antibiotic, cefixime is often prescribed to combat bacterial infections of the throat, ear, urinary tracts and lungs, as well as gonorrhoea, pneumonia and bronchitis [5,66]. The results of their study suggested that the antibacterial activity of the cefixime rose by eight times when integrated with AuNPs. Additionally, cefixime’s effective treatment period of eight hours was reduced to half upon integration with AuNPs. They further evaluated the cytotoxicity associated with the prepared NPs. Up to a concentration of 75 μg/mL, cefixime conjugated-AuNPs did not exhibit notable cytotoxic effects on human cells. On the other hand, as dosage increased to 100 μg/mL, a reduction in cell viability by approximately 0.50% was noted. The mechanism for the enhanced antibacterial activity of the cefixime-integrated AuNPs can be summarized in three steps. The initial step involves the interaction of the cefixime with AuNPs to form cefixime-stabilised AuNPs. As the metal NPs are highly responsive to sulphur, the synthesised NPs then interact with the bacterial cell wall by interacting with the protein and DNA through the sulphur as well as the phosphorous atom. Finally, the release of the AuNPs into the cellular matrix leads to cell lysis causing damage as well as the death of bacterial cells. They have presumed that the cefixime has enhanced the membrane’s permeability through interaction with the peptidoglycan barrier of *S. aureus*, leading to the well penetration of AuNPs to the bacterial cell facilitating the interaction with protein and DNA and causing cell death [5].

The formation of biofilms is an important approach through which bacteria can also develop antibacterial resistance. Bacterial biofilm comprises a complicated microbial cluster encased by an array of extracellular polymeric compounds that are produced while a community of microbes clings irrevocably to a static or living substrate [6]. However, not all biofilms are surface based. For example, there are wound biofilms and respiratory biofilms. Besides the assistance provided by the polymeric array, microorganisms in biofilms may execute a variety of survival tactics to avoid diagnosis by the host mechanisms of defence. This framework is regarded as a major virulence parameter since it is challenging to eliminate and is usually attributed to therapeutic troubles. The bacteria across the biofilm demonstrate changes in metabolism, protein synthesis and gene transcription in response to ambient anoxia as well as nutritional limitation. This can result in reduced metabolic activity and a slower rate of cell replication [6,67]. The biofilm-related illnesses are often chronic infections that emerge slowly, are hardly cured by the body’s immune system, and show inconsistent responses to antimicrobial therapies. Due to its wide range of activity, photodynamic antimicrobial therapy (PACT) comes out as a promising and effective therapeutic remedy for multidrug-resistant bacterial biofilm. *E. faecium*, *K. pneumoniae*, *S. aureus*, *P. aeruginosa*, *Enterobacter* and *A. baumannii* are some of the bacterial strains that are very prone to biofilm formation [62]. This leads to the development of resistance against common antibiotics like β-lactams, tetracyclines, macrolides, fluoroquinolones and aminoglycosides, etc. The greater surface area attributed to an increased surface-to-volume ratio improves the penetrations as well as associations of the NPs with the biofilm network in comparison with pure drug molecules. AuNP have been extensively studied as photosensitisers owing to their unique attributes, such as their excellent biocompatibility in human cells, smaller size optical characteristics and simplicity of functionalisation through the surface ligands, which render them fascinating for a wide range of applications, particularly PACT [6,68]. Nowadays, antimicrobial therapy, which involves the use of photoactive substances, has appeared as an excellent alternative to combat the infections caused by antibiotic-resistant bacteria. *P. aeruginosa* has developed resistance against several widely employed antibiotics, such as β-lactams, quinolones, aminoglycosides and cephalosporins. Furthermore, metal NPs minimise antibiotic dosages as well as the side effects [69].

In 2020, Rocca et al. prepared gold AuNPs coated with a lower concentration of amoxicillin as an alternative for PACT and employed them as a photoactive compound for the reduction of the biofilm produced by *P. aeruginosa* (PAE 189718 and PAE 191150) and *S. aureus* (MRSA 773 and MRSA 771) bacteria [6]. Upon irradiating them with the amoxicillin-coated AuNPs (1.5 μg/mL) in the presence of white light, the viability of the *S. aureus* bacteria in the biofilm reduced to 60%, while a 70% reduction was observed in the case of *P. aeruginosa* biofilms after 3 h. However, no changes in the viability of the *S. aureus* biofilm were reported when treated with pure amoxicillin, indicating the high antibacterial resistance of *S. aureus* against amoxicillin. The AuNPs were synthesised using a single-step synthetic procedure by employing amoxicillin trihydrate as a reducing and capping agent. The biofilms were developed in 96 well-plates. Further, they reported that when the *S. aureus* biofilms were treated with a pure amoxicillin concentration 10 times higher than the MIC of amoxicillin-coated AuNPs, they did not show any reduction in the viability of bacterial stain. Penetration within biofilm, as well as microbial adhesion, may be greatly influenced by electrostatic attraction among the negatively charged extracellular matrix of the biofilm and the positively charged amoxicillin-coated AuNPs. Furthermore, the localised surface plasmon resonance exhibited by AuNPs produces a specific heating on the surface of the particles. Thus, the bacteria near the particle’s surface may suffer cellular damage as a result of the increased temperature, which might eventually result in the death of the bacteria [6]. While combating biofilm formation, the primary goal is the eradication of either a 5-log reduction or sometimes a 3-log reduction. Since this study reported a 60% and 70% reduction in the viability of *S. aureus* and *P. aeruginosa* biofilms, it gives room for further improvement in the activity of the prepared amoxicillin-coated AuNPs.

In another study, Halawani et al. developed amoxicillin-conjugated AuNPs to combat the multidrug resistance developed by *E. coli* and the methicillin-resistant *S. aureus* bacteria [59]. Amoxicillin is classified as a β-lactam antibiotic, which is typically utilised for the treatment of microbial infections of the urinary and respiratory tracts. It can prevent the formation of bacterial cell walls and is efficient against numerous types of antibiotic-resistant bacteria [59,70]. They have isolated the bacterial strains from several infection sources and identified them through biotype, phenotype and 16S rRNA gene evaluations. For the synthesis of the AuNPs, aqueous *Juniperus excelsa* leaf extract was used. Sodium tri-polyphosphate (TPP) was employed as a linker component for the fabrication of the amoxicillin-conjugated gold nanodrug. They have reported that the increase in the volume of the extract makes the absorption spectra more intense and sharper, indicating the increase in the concentration of AuNPs with decreased size. This indicates that a higher concentration of extract was suitable for the synthesis of AuNPs. They have also reported that smaller size of the NPs exhibits a greater concentration of amoxicillin and show enhanced conjugation potential. This is due to the higher surface area associated with the small size of the NPs. As the size increases, the conjugation efficiency decreases [59]. They have evaluated the antibacterial activity of the pure AuNPs and amoxicillin-conjugated AuNPs using agar well diffusion method and reported that the synthesized AuNPs do not exhibit any zone of inhibition, indicating the absence of antibacterial activity. The amoxicillin-conjugated AuNPs exhibit an inhibition zone ranging from 20 to 37 nm. The MIC associated with amoxicillin was 96–114 µg/mL. However, in the case of amoxicillin-conjugated AuNPs, the MIC was reduced by 12–31 times, i.e., 3.6–8 µg/mL. The TEM analysis reveals the presence of the amoxicillin-conjugated AuNPs in the cell wall as well as inside the bacterial cell. The antibacterial activity depends upon the release rate of amoxicillin from the NPs inside the cell. AuNPs enhanced the penetration and release of the antibiotic molecules inside the cell. However, amoxicillin alone is unable to penetrate the bacterial cell. Amoxicillin-conjugated AuNPs destroy the bacterial cell wall by inhibiting the production of mucopeptide caused by amoxicillin. After penetrating through bacterial cell walls, it interferes with the phosphorus present in RNA or DNA causing disruption in protein synthesis, which leads to cell lysis [59]. Here, the AuNPs serve as drug carriers and delivery systems for amoxicillin to combat bacterial infections.

Fenniri and coworkers, in another study, prepared ampicillin-conjugated AuNPs decorated on PEG-functionalised rosette nanotubes to enhance the antimicrobial activity of ampicillin [58]. They investigated the antimicrobial activity against *S. aureus* as well as MRSA bacteria. The MIC value for *S. aureus* bacteria was reported as 0.58 μg/mL, which was 18% less than the MIC value of pure ampicillin. However, in the case of MRSA bacteria, the MIC was reduced by 10–20 times as compared to the ampicillin antibiotic, revealing significant enhancement in the antibacterial activity. The MIC of ampicillin against MRSA was reported as 32 to 50 µg/mL. However, for the ampicillin-conjugated AuNPs, the MIC value was 4 µg/mL against MRSA, which is almost ten times lower than the ampicillin alone. The electrostatic attraction between the bacterial cell wall, which bears a negative charge, and the cationic behaviour of the prepared AuNP system enhances the binding potential of the antibiotics with the bacterial cell wall thereby facilitating the release of the antibiotic drug molecules. However, AuNPs themselves can interfere with the bacterial metabolic system. Ampicillin, a β-lactam antibiotic, works by specifically targeting proteins that bind to penicillin. These are essential for the formation of bacterial peptidoglycan, required for the constitution of bacterial cell walls. The synergistic effects of these three mechanisms contribute to the enhancement of the antibacterial activity of the ampicillin-conjugated AuNP system.

Kaur et al. prepared different AuNPs using trisodium citrate, Tween 20 and polyvinylpolypyrrolidone as surfactants using chemical methods [60]. These prepared NPs were then coated with amikacin to gain superior antimicrobial activity. The formation of AuNPs was confirmed through UV-visible absorption spectroscopy. As the size of the surfactant molecule decreases, the amount of amikacin loaded in the AuNPs increases. They reported the density of citrate, Tween 20 and polyvinylpolypyrrolidone-capped AuNPs as 1.78 ×10^14^/mL, 2.4 × 10^13^/mL and 4.2 × 10^12^/mL, respectively. For this reported density, the amount of amikacin loaded on the AuNPs synthesised using citrate, Tween 20 and polyvinylpolypyrrolidone as surfactants were 60 µg/mL, 46 µg/mL and 40 µg/mL, respectively. They evaluated and compared the antibacterial activity of the prepared AuNPs, pure amikacin and amikacin-coated AuNPs against *E. coli* and *S. aureus* bacteria using the agar well diffusion method. Pure AuNPs do not exhibit any antimicrobial properties. However, the antibiotics-coated AuNPs demonstrated a higher zone of inhibitions against both the bacterial strain in the antibacterial activity test as compared to amikacin, indicating enhanced antimicrobial activity. For the *E. coli* and *S. aureus* bacterial strains, amikacin alone demonstrated an inhibition zone ranging from 7 to 8 mm and 5 to 6 mm, respectively, depending on different concentrations. However, for the same concentrations, the antibiotic-coated AuNPs demonstrated a higher zone of inhibitions, ranging from 10 to 13 mm for *E. coli* bacteria and 6 to 8 mm for *S. aureus* bacteria. Regarding the mechanism of action of the prepared NP system, they reported that since gold serves as a soft acid, the AuNPs have a great potential to interact readily with soft bases like the phosphorus and sulphur that exists in DNA, as well as the proteins of the bacterial cell membrane, leading to cell lysis or the death of bacteria. The cationic behaviour of amikacin tends to interact with the anionic substances of the bacterial strain, thereby inhibiting protein synthesis through adhering with the 30S ribosomal subunit [60].

A wide range of Gram-negative bacterial strains secrete ESBL enzymes which display enhanced resistance against frequently employed antibiotics like penicillins and cephalosporins. Cefotaximase-Munich (CTX-M) is one such common ESBL found in *K. pneumoniae* and *E. coli* bacteria [61]. However, among different varieties of CTXM, the most prevalent one is CTXM-15, which is found extensively in India. These bacterial strains have developed resistance against cefotaxime. Thus, to enhance the antibacterial activity of cefotaxime antibiotics, Shaikh et al. conjugated it with biosynthesized AuNPs and evaluated the antibacterial activity against the cefotaxime-resistant *E. coli* and *K. pneumoniae* bacterial strain [61]. The AuNPs were prepared using a bio enzyme, bromelain, as a reducing and capping agent. The loading ability of the prepared AuNPs for cefotaxime was reported to be 77.59%. The MIC value for cefotaxime-coated AuNPs was reported as 2.018 μg/mL and 1.009 μg/mL against the *K. pneumoniae* and *E. coli* strains, respectively. Furthermore, the MBC value was reported as 4.037 μg/mL and 2.018 μg/mL, respectively, against *K. pneumoniae* and *E. coli*. The synthesised AuNPs alone do not exhibit any antibacterial activity. The conjugation with AuNPs increases the release rate of the antibiotics in the bacterial cell, thereby increasing the activity.

In another study, Hagbani et al. used AuNPs as a vehicle to deliver as well as to increase the antibacterial activity of vancomycin [62]. The antibacterial property of vancomycin is often associated with its potential to impede the formation of the cell walls of bacteria by adhering to a particular C-terminal sequence, D-Ala-D-Ala, within the peptidoglycan pentapeptide [71]. During the synthesis of vancomycin-coated AuNPs through a one-pot synthesis method, the antibiotics themselves play the role of capping and reducing agents. The zeta potential analysis of the coated AuNPs demonstrated a high negative value of −18 meV indicating high stability of the prepared vancomycin-coated AuNPs. The drug loading efficiency of the AuNPs was reported to be 86.2% for the evaluation of antibacterial activity, and a comparative study was carried out between the pure vancomycin and vancomycin-loaded AuNPs against the Gram-positive *S. aureus* and Gram-negative *K. oxytoca*, *E. coli* and *P. aeruginosa* bacterial strains. Although both pure vancomycin and the vancomycin-coated AuNPs show antibacterial activity, the effective concentration of vancomycin was reduced by half when conjugated with AuNPs. Here, AuNPs acts as a drug carrier as well as contributing to the enhancement of antibacterial activity by hindering the bacteria ATPase functions and disrupting the potential of the membrane. This combined effect of the vancomycin and the AuNPs results in structural damage, inadequate surface adherence, disruption of metabolic processes and, eventually, bacterial cell death.

Another widely used antibiotic against which various Gram-negative bacteria have developed resistance is cefoxitin. Cefoxitin falls under the second-generation cephalosporin class of antibiotics [32,72]. Nowadays, this drug is losing its effectiveness because of the alteration of porins, including in numerous Gram-negative bacteria. The resistance acquired by *K. pneumoniae* and *E. coli* strains is due to the alterations in the proteins ompK36 and ompK35 in the outer membrane [72]. Porins usually create small channel-like structures in the outer membrane through which small hydrophilic compounds as well as β lactam antibiotics may migrate [51]. However, the modification of the porins hinders the diffusion of the drug molecules. Further, AuNPs have the ability to interact with the bacterial cell membrane and create pores causing the disruption of the cell wall. Hence, cefoxitin, when conjugated with AuNPs, can carry and release the drug molecules in the bacterial cell more efficiently even if the porins disappear or are altered. To revive the potency of the cefoxitin drug, Rizvi and coworkers conjugated it with AuNPs using cefoxitin itself as the reducing and capping agent [32]. The prepared antibiotics-coated AuNPs exhibit higher stability indicated by the high negative zeta potential value of −23.6 ± 1.6 mV with a drug loading efficiency of 71.92%. No additional cross-linking agent was employed during the synthesis of cefoxitin-loaded AuNPs. They have evaluated the antimicrobial activity of the prepared cefoxitin-coated AuNPs against Gram-negative *K. pneumoniae* and *E. coli* strains, which have developed resistance against cefoxitin following the well diffusion method. Surprisingly, cefoxitin alone does not display any antibacterial activity against both bacterial strains up to a concentration of 6 μg/well, while cefoxitin-coated AuNPs demonstrate an inhibition zone of 18 mm and 17 mm against the *E. coli* and *K. pneumoniae* bacterial strains at the same concentration. On conjugation with AuNPs, the antibacterial property was significantly enhanced and the MIC_50_ value was reported as 2.5 µg/mL and 1.5 µg/mL, respectively, for *K. pneumoniae* and *E. coli* bacteria. However, cefoxitin alone possesses a MIC_50_ value of 19.5 µg/mL and 23 µg/mL, respectively, for *E. coli* and *K. pneumoniae* bacteria.

Haddada et al. fabricated doxycycline-coated AuNPs and evaluated the antibacterial activity against Gram-positive *S. aureus*, *E. faecalis* and *E. faecium* bacteria as well as against Gram-negative *E. coli*, *K. pneumoniae*, *P. aeruginosa* and *A. baumannii* bacteria [11]. The chemical structure of doxycycline possesses a tetracyclic unit with aromatic hydroxyl anthraquinone rings that serve as chromophores contributing to the therapeutic effect [11,73]. PEG was also used as a stabilising agent during the synthesis of AuNPs. The AuNPs showed a maximum drug loading efficiency of 86%. Pure doxycycline exhibited a MIC value of ~32 μg/mL against *K. pneumonia*, *P. aeruginosa*, *E. faecalis*, *S. aureus* and *E. faecium* bacterial strains, which were reduced to 2 μg/mL when doxycycline-coated AuNPs were employed, indicating a significant enhancement in antimicrobial properties. However, for *E. coli* bacteria, they reported that pure doxycycline as well as doxycycline-coated AuNPs exhibited a MIC value of 2 μg/mL. The detailed explanation regarding the antimicrobial activity against *E. coli* bacteria was not provided in the manuscript.

Yang and coworkers prepared AuNPs coated with pharmaceutical intermediate compounds, including 7-aminodesacetoxycephalosporanic acid (7-ADCA), 7-aminocephalosporanic acid (7-ACA) and 6-aminopenicillanic acid (APA), and evaluated the activity towards MDR bacterial strains isolated from a patient’s saliva or urine [63]. They referred to these compounds as “antibacterial intermediates”, as they comprise the major structural unit of β-lactam antibiotics. The conjugation of different pharmaceutical intermediates which are used for the chemical synthesis of antibiotic drugs with NPs has appeared as an innovative approach to fight against antimicrobial resistance. AuNPs coated with such pharmaceutical intermediate also possess the ability to kill MDR as well as Gram-negative bacterial strains [63]. These coated AuNPs have been reported to be effective against Gram-negative bacteria, while for Gram-positive bacteria they are reported to be inactive because of the higher thickness of the cell wall, which may hinder the penetration of the NP system. They reported that the 6-aminopenicillanic acid (APA)-coated AuNPs demonstrated superior antibacterial properties compared to other antibacterial intermediates. From the toxicity study, they reported that the prepared AuNPs conjugated with APA are highly biocompatible and do not impart any toxic effect to human cells up to a concentration of 20 μg/mL, which is almost eight times higher than the MIC value against *E. coli* and *K. pneumoniae* and four times compared to the MIC values against *P. aeruginosa* as well as MDR *E. coli* and *K. pneumoniae*. The MIC value of APA-coated AuNPs was 5 μg/mL for *P. aeruginosa* and MDR *E. coli* and *K. pneumoniae*. For *E. coli* and *K. pneumoniae*, the MIC value was reported as 2.5 μg/mL. However, the MIC value of the 6-aminopenicillanic acid molecule alone was greater than 250 μg/mL.

Due to the growth of pathogens that have evolved resistance to all existing conventional antibacterial medications, colistin is being employed as a therapeutic option once again. Colistin is used as a last-line antibiotic to combat both acute and chronic illnesses caused by Gram-negative bacteria. A higher dose of colistin imparts numerous side effects to human health [64,74]. Hence, dose reduction is essential for the enhancement of the therapeutic effect of the drug. In this regard, Fuller et al. fabricated colistin-coated AuNPs to reduce the dose of the drug and evaluated the antibacterial activity against the Gram-negative *E. coli* bacterial strain [64]. The prepared colistin-coated AuNPs demonstrated a 6.8 times reduction in the MIC value as compared to pure colistin.

Carbapenems are a type of highly efficient antibiotic that is frequently employed for curing fatal bacterial infections. Carbapenems are broad-spectrum antibiotics and are usually susceptible to hydrolysis via numerous β-lactamases, operate as inhibiting agents of β-lactamases or “slow substrates” in certain cases and assault penicillin-binding proteins, which makes them differ from other β-lactam antibiotics [65,75]. However, the emergence of antibiotic-resistant bacteria in recent times has drastically reduced its clinical significance. Shaker et al. prepared carbapenem-coated AuNPs using meropenem and imipenem and demonstrated the antibacterial activity using Gram-negative carbapenem-resistant *K. pneumoniae*, *P. mirabilis* and *A. baumanii* bacterial strains isolated from infected patients [65]. They synthesised AuNPs possessing particle sizes 35, 70 and 200 nm, with the best results reported being particle sizes of 35 nm. Carbapenems can be easily functionalised, preventing any chemical alteration of the drug molecules because of the ample interactions of the thioether group of the meropenem and imipenem molecules with AuNPs. The surface-to-volume ratio associated with the smaller size of the NPs enhances the drug-loading potential, thereby increasing the antibacterial activity of the carbapenems. A maximum of ~73% drug-loading ability was reported for AuNPs with a particle size of 35 nm. However, with an increase in the particle size, the loading efficiency of the drug decreases. A minimum drug loading of ~41% has been reported for AuNPs with a particle size of 200 nm. However, drug release efficiency was also associated with the particle size of the NPs. The small size of the NPs showed a faster drug release rate. A total of 89% and 94% of drug releases were reported for AuNPs with a particle size of 35 nm. Imipenem-coated AuNPs with a particle size of 35 nm decrease the MIC value by three and four times against *P. mirabilis* and *A. baumanii* as well as *K. pneumoniae* bacteria, respectively. However, meropenem-coated AuNPs reported two times and three times, respectively, for *P. mirabilis* and *A. baumanii* bacteria. Pure AuNPs do not exhibit antimicrobial properties against the tested bacterial strains. The carbapenem-loaded AuNPs interact with the cell membrane of the bacterial strain, and as a result, the loaded drug molecules may conjugate with the penicillin-binding proteins and accumulate in the outer cell membrane of the bacteria. This integration of the drug molecules can result in the disruption of the integrity of the bacterial cell wall or may impede the formation of the cell wall of the bacteria causing the death of the bacterial strain.

## 6. Antibacterial Flavonoids and Heterocyclic Compounds Coated AuNPs to Combat AMR

**Figure 3:**
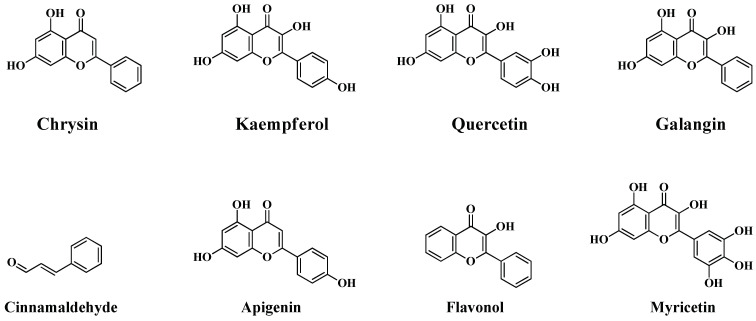
Chemical structure of some flavonoids possessing antibacterial activity.

Flavonoids are a class of bioactive natural compounds originating from plants, that exhibit a wide range of medicinal properties, including antibacterial ones [76]. These are the secondary metabolites comprising a benzopyrone ring possessing phenolic groups [77]. The structures of some flavonoid compounds that have been reported to bear antimicrobial properties are depicted in Figure 3. It has been reported that the inhibitory efficacy of flavonoids against Gram-positive bacteria was greater than that against Gram-negative bacteria [78]. Alhadrami et al. took the advantages associated with the AuNPs, including excellent drug carrier properties and high polyvalent effects [76]. They analysed the antibacterial activity of several pure flavonoids and found that kaempferol and quercetin possess superior antimicrobial properties towards Gram-negative bacteria, hence they were considered for conjunction with AuNPs along with chrysin, which exhibits superior antibacterial properties against Gram-positive bacteria. They have prepared the AuNPs using L-glutathione through a chemical synthesis method. The carboxylate terminal of the glutathione exhibits an excellent binding ability towards flavonoids, thereby facilitating the conjunction with AuNPs, which increases stability, and their hydrophilic nature increases the water solubility of AuNPs. The conjunction efficiency of the flavonoids was reported as 41, 71 and 80%, respectively, for chrysin, kaempferol and quercetin. The antibacterial activity of the coated AuNPs was examined against the Gram-negative *E. coli*, *P. aeruginosa*, *K. pneumonia* and *P. vulgaris* bacterial strains. Among the three flavonoids, quercetin-coated AuNPs were reported as the most active antibacterial agent, possessing a MIC value of 30 µg/mL for *E. coli*, *P. aeruginosa* and *P. vulgaris* and 60 µg/mL for *K. pneumonia*. They reported that quercetin and kaempferol alone possesses a MIC value of 0.48 mM against *P. vulgaris*, *K. pneumonia* and *P. aeruginosa* bacteria. However, for *E. coli* bacteria, it was reported as 0.24 mM. Chrysin alone possesses a MIC value > 1 mM. The interaction of the flavonoids-coated AuNPs with bacterial cell walls increases the rigidity of the cell membrane, thereby decreasing the functionality. Flavonoids also possess the ability to inhibit the functions of DNA gyrase of *E. coli* bacteria possessing the IC_50_ value between 0.9 and 3.9 µM.

In another study conducted by Ramasamy et al., cinnamaldehyde, a natural flavonoid compound possessing antibacterial activity was employed to coat AuNPs to impart enhanced antibacterial properties [79]. The prepared flavonoid-coated AuNPs were found to be efficient for the eradication of bacterial biofilms formed by Gram-negative *E. coli* and *P. aeruginosa* as well as Gram-positive *S. aureus* and MRSA bacteria. They used tyrosine during the AuNPs’ synthesis, which acts as the reducing as well as the capping agent. Further, the surface functionalisation of AuNPs was performed using Tween 80 and TEOS. The prepared flavonoid-coated AuNPs exhibited two peaks at 540 nm and 290 nm in UV-visible spectroscopy for the AuNPs and the cinnamaldehyde molecules. The flavonoid-conjugated nanoformulation with a cinnamaldehyde concentration of 0.005% (*v*/*v*) was reported as effective for the inhibition of bacterial biofilm formation, as a greater than 50% inhibition in the biofilm formation was obtained at such concentration. However, for cinnamaldehyde alone, the effective concentration for reducing the biofilm formation was reported as 0.025% (*v*/*v*), indicating the increased activity of cinnamaldehyde-coated AuNPs.

Riaz et al., on the other hand, developed a green synthetic approach for the fabrication of flavonoid-coated AuNPs [80]. Flavonoids were extracted from the methanolic leaf extract of *Berberis lyceum* and used for the stabilisation of AuNPs during synthesis. The prepared coated AuNPs showed antibacterial activity against the *E. faecalis* bacterial strain, with a MIC value of 25 µg/mL.

**Figure 4:**
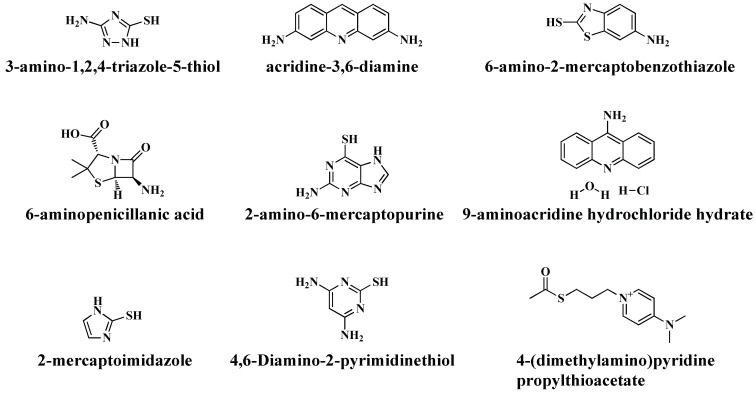
Chemical structure of some heterocyclic compounds possessing antibacterial activity.

Moreover, the chemical structures of some small heterocyclic compounds that have been successfully conjugated with AuNPs to fight against AMR are represented in Figure 4 [81,82,83,84,85]. 

Table 3 shows few examples of heterocyclic compounds-coated AuNPs and their activity against different bacteria. In 2020, Zhang et al. developed a new strategy for the treatment of oral *P. gingivalis* through the coating of aligners with the heterocyclic compound 4,6-diamino-2-pyrimidinethiol-conjugated AuNPs [81]. The synthesized nano formulation was reported as efficient for the inhibition of the growth of bacteria as well as for eradicating biofilm formation. From the biological safety aspects, they reported that the prepared nano formulation did not impart any toxic effect on the human umbilical vein endothelial cells (HUVEC) at a dose below 100 g/mL. The concentration of the 4,6-diamino-2-pyrimidinethiol-conjugated AuNPs on aligner coating was 1.109 ± 0.021 μg/cm^2^, and the incubation concentration was reported as 517 ± 12.6 μg/mL. The bacterial growth was diminished when the concentration of the bacterial suspension was 10^4^ CFU/mL (colony-forming units per mL). Increasing the concentration of the bacterial suspension to 10^5^ CFU/mL bacterial growth slowed initiation. Thus, at the concentration of 1.109 ± 0.021 μg/cm^2^, the conjugated AuNPs were effective in inhibiting the growth of the bacteria up to the bacterial suspension concentration of 10^4^ CFU/mL.

Acridines are heterocyclic organic compounds containing one N atom possessing enormous pharmacological properties. Because of their ability to interact strongly with biomolecules, including protein and DNA, they are widely employed in biological fields [86,87]. Acridine-based therapies are widely recognised for their immense antimalarial, antifungal and antibacterial properties [82]. Considering these significant pharmacological properties, Basu and coworkers developed AuNPs coated with acridine derivatives, including 9-aminoacridine hydrochloride hydrate, acridine orange, proflavine and acridine yellow dyes [82]. Citrate was employed for the stabilisation of the NPs. The amine groups on the medicines may enable covalent bonding with the AuNPs. The antibacterial activities of the pure drug molecules as well as the drug-coated AuNPs were evaluated against *B. subtilis* and *E. coli* bacteria and it was reported that 9-aminoacridine hydrochloride hydrate and acridine orange demonstrated antibacterial activity alone as well as when conjugated with AuNPs. Conjugation of the heterocyclic compounds with AuNPs enhanced the antibacterial activity of the molecules. However, no antibacterial property was detected for pure proflavine and acridine yellow and their conjugated AuNPs. One AuNP was capable of adsorbing many pharmacological molecules; hence, with the increase in AuNP concentration, the antibacterial property also increases. The interaction of the coated AuNPs with the cell wall disrupts the membrane structure, creating pores through which cause the cellular contents to leak or explode, resulting in an osmotic imbalance, cellular integrity loss and, ultimately, cell death.

In another study, Wang et al. prepared AuNPs coated with small N-heterocyclic compounds that included 3- amino-1,2,4 triazole-5-thiol, 6-amino-2-mercaptobenzothiazole, 2-amino-6-mercaptopurine, 6-aminopenicillanic acid and 2-mercaptoimidazole [83]. The heterocyclic compounds as well as pure AuNPs did not demonstrate any antimicrobial activities against the tested pathogenic bacteria. When conjugated with AuNPs, all heterocyclic compounds, except 6-aminopenicillanic acid, revealed immense antibacterial properties. All the coated AuNPs reduced the viability of the *S. aureus* biofilm. 3-Amino-1,2,4 triazole-5-thiol- and 6-amino-2-mercaptobenzothiazole-coated AuNPs showed a 2.5-log reduction in the viability of *S. aureus* biofilm, 2-mercaptoimidazole-coated AuNPs showed a 5-log reduction. However, 2-amino-6-mercaptopurine- and 2-mercaptoimidazole-coated AuNPs showed a 2-log reduction in the viability of *E. coli* biofilm. They assumed that, except for 6-aminopenicillanic acid-conjugated AuNPs, all other conjugated AuNPs exhibit a positive charge, making it feasible to attack the bacterial cell wall carrying negative charges. 2-mercaptoimidazole-coated AuNPs exhibited enhanced biocompatibility and did not impart any toxic effect up to a concentration of 100 mg/mL. Among all the nanoformulations, 2-mercaptoimidazole- and 3-amino-1,2,4-triazole-5-thiol-coated AuNPs demonstrated superior antibacterial activity against *E. coli* and *S. aureus* bacteria as well as their MDR variants. These two AuNPs also exhibit activity against bacterial biofilm reduction.

Li et al. developed 4,6-diamino-2-pyrimidinethiol-coated AuNPs to serve as a useful alternative to cure disease caused by the *E. coli* bacterial strain [84]. They evaluated the toxicity of the synthesised nanoformulation by assessing the impact of the long-term delivery of the coated AuNPs to mice. While uncoated AuNPs disrupted the mitochondrial membrane, the coated AuNPs had no effect on the intestinal epithelial cells’ mitochondria. A biocompatibility study demonstrated that the viability of intestinal epithelial cells (MODE-K) was 16.5% greater as compared to pure AuNPs. They reported that the 4,6-diamino-2-pyrimidinethiol-coated AuNPs are more biocompatible with intestinal epithelial cells (MODE-K), and the number of dead cells in the case of the coated AuNPs was 60% lesser than that in the case of pure AuNPs. The antibacterial activity of the prepared AuNPs was evaluated against *E. coli* bacteria in comparison with pure AuNPs and levofloxacin, and it was reported that the number of living bacteria decreased by 85.2% and 91.2%, respectively, in comparison to pure AuNPs and coated AuNPs, indicating their higher effectiveness.

**Figure 5:**
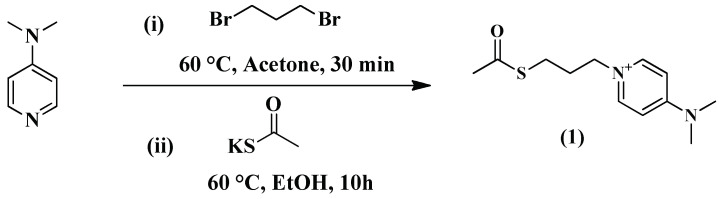
Scheme for the synthesis of 4-Dimethyl aminopyridinium propylthioacetate.

A novel aminopyridine-based heterocyclic compound, 4-Dimethyl aminopyridinium propylthioacetate (1), which acts as a cationic ligand, was prepared by Anwar and coworkers (Figure 5) and conjugated with AuNPs to obtain immense antibacterial properties [85]. The antimicrobial activity of the prepared nanoformulation was compared with pure 4-Dimethyl aminopyridinium propylthioacetate as well as with the antibiotic pefoxacin against the Gram-negative *E. coli* bacterial strain. The reported reaction scheme for the synthesis of 4-Dimethyl aminopyridinium propylthioacetate is represented below.

The MIC values for the pefloxacin antibiotic, 4-Dimethyl aminopyridinium propylthioacetate and their coated AuNPs were reported as 50 µg/mL, 300 µg/mL and 200 µg/mL. This indicates that incorporation with AuNPs significantly increases the antibacterial activity of the prepared heterocyclic compound. Further mixing of the coated AuNPs with pefloxacin also showed an enhancement in the antimicrobial activity, thereby reducing the MIC value up to 25 µg/mL, which is almost half compared to the pure antibiotics.

## 7. Conclusions

AuNPs are effectively progressing toward clinical trials. The small particle size offers a large surface area which enhances drug-loading efficiency as well as a higher penetration power of the NP through the bacterial cell wall, which makes them an efficient tool for drug delivery. Among numerous metal and metal oxide NPs, pure AuNPs or those conjugated with antibacterial agents or drugs emerge as fascinating because of the easy-to-synthesise, nontoxic nature and high biocompatibility of Au. Apart from improved delivery, numerous studies have shown that combining AuNPs with antibacterial drugs increase antibacterial properties while minimising side effects through decreasing the antibiotic dosages. Still, the mechanical intricacies of its activity, cytotoxicity and the biosafety associated with the AuNPs are the areas that necessitate special and detailed research. Moreover, although the mode of action of the antibiotics is well known, the mechanism underlying the antibacterial activity of the antibiotic-conjugated AuNPs is less clear. Hence, a systematic investigation to evaluate the proper mechanism of antibacterial action associated with conjugated AuNPs is very important. Special attention should be given in the large-scale preparation, cost effectiveness and safety of antibiotic-coated AuNPs. In the current scenario of antibiotic resistance, transforming an inept antibiotic into a robust nanoformulation is a delightful concept. However, only a few studies have reported on the synthesis of antibiotic-conjugated AuNPs utilising green methods, leaving room for further research in this area.

## Figures and Tables

**Table 1:** Examples of multidrug resistant pathogenic bacteria and their harmful effect on human health.

Name of the Bacteria	Type of the Bacteria	Resistance to Antibiotics (Classes)	Infections Caused	Refs.
*Staphylococcus aureus*	Small round-shaped, Gram-positive bacteria	Methicillin, vancomycin, tetracycline, quinolone, fluoroquinolones, penicillin, phenicol, lipopeptide, aminoglycosides	Nosocomial infections, soft tissue infections, endocarditis, pneumonia, suppurative diseases, respiratory tract infections, fatal sepsis.	[42]
*Escherichia coli*	Rod-shaped, Gram-negative bacteria	Carbapenems, aminoglycosides, β-lactams, quinolone, polymyxins	Urinary tract infections, hospital-acquired infections.	[9]
*Klebsiella pneumoniae*	Rod-shaped, Gram-negative bacteria	β-Lactams, carbapenems, aminoglycosides, rifamycins	Pneumonia, urinary tract infections, bloodstream infections, skin infections.	[9]
*Klebsiella oxytoca*	Rod-shaped, Gram-negative bacteria	Aminoglycosides, cephalosporin, carbapenems, fluoroquinolones, tetracycline, chloramphenicol	Bacteremia, antibiotic-associated hemorrhagic colitis, mild diarrhoea, urinary tract infections, bloodstream Infections.	[43]
*Pseudomonas aeruginosa*	Rod-shaped, Gram-negative bacteria	β-Lactams, carbapenems, fluoroquinolones, aminoglycosides, quinolone	Nosocomial infections, urinary tract infections, pneumonia, skin infections, chronic lung disease like cystic fibrosis.	[44]
*Acinetobacter baumannii*	Short rod-shaped, Gram-negative bacteria	Carbapenem, imipenem, aminoglycosides, Fluoroquinolones	Urinary tract infections, bloodstream infections, hospital- acquired infections.	[9]
*Proteus mirabilis*	Rod-shaped, Gram-negative bacteria	Penicillin, folate inhibitors, quinolone	Gastrointestinal tract infections, crohn’s disease, respiratory tract infections, skin infections, bloodstream infections.	[45]
*Enterococcus faecalis*	Slightly oval shaped, Gram-positive bacteria	Glycopeptides, oxazolidinone, lipopeptide	Abdominal infections, pelvic infections, septicaemia.	[46]
*Porphyromonas gingivalis*	Rod-shaped, Gram-negative bacteria	Tetracyclines, penicillin, lincosamides, nitroimidazole	Periodontal disease.	[47]
*Micrococcus luteus*	Sphere-shaped, Gram-positive bacteria	Macrolides, cephalosporin, penicillin	Hepatic and brain abscess, bacteremia, bloodstream infections.	[48]
*Aeromonas hydrophila*	Rod-shaped, Gram-negative bacteria	Penicillin, ampicillin, tetracycline	Bacteremia, diarrhoea, gastroenteristrics, urinary tract infections.	[49]
*Staphylococcus epidermidis*	Grape-like clusters shaped, Gram-positive bacteria	Vancomycin, glycopeptides, rifamycins	Bloodstream infections, surgical site infections.	[50]

**Table 2:** Some recently developed antibiotic-coated AuNPs and their activity towards combating AMR.

Name of Antibiotics	Class of Antibiotics	Size and Shape of AuNPs	Time, Temperature of Synthesis and UV Absorption Peak of AuNPs	Tested Bacteria	MIC	Remarks	Refs.
Cefixime	Cephalosporin	Spherical, size ranging from 25 to 50 nm.	2.5 h 532 nm	*S. aureus*	45 ± 0.12 μg/mL (correspond to 3.24 μg of cefixime)	Efficiency of cefixime increased by 8 times when conjugated with AuNPs.	[5]
Amoxicillin	Penicillin	Irregular shapes including triangular, hexagonal, spherical, etc.	18 min and 50 °C	*P. aeruginosa*, *S. aureus*	1.5 µg/mL	A 60% and 70% reduction in the viability was obtained for *S. aureus* and *P. aeruginosa* biofilm, respectively.	[6]
Amoxicillin	Penicillin	Hexagonal and spherical shape, size between 15.99 and 24.71 nm.	1 h and 25 °C 534 nm	MRSA, *E. coli*	3.6–8 µg/mL	The synthesised coated AuNPs exhibited a MIC 12–31 times less compared to pure amoxicillin.	[59]
Ampicillin	Penicillin	1.43 ± 0.5 nm.	24 h and room temp	MRSA, *S. aureus*	0.58 μg/mL against *S. aureus* and 4 μg/mL for MRSA	The MIC reduced by 18% against *S. aureus* bacteria and 10–20 times against MRSA as compared to ampicillin alone.	[58]
Amikacin	Aminoglycoside	All possesses spherical morphology, for citrate- AuNPs, the average particle size is 3.3 nm, for PVP-AuNPs average size is 11.5 nm and for Tween 20-AuNP average size is 6.25 nm.	2 h and room temp. 533, 537 and 535 for citrate, Tween 20 and PVP capped AuNPs, respectively.	*E. coli*, *S. aureus*	-	All the amikacin-coated AuNPs fabricated using different surfactants exhibit enhanced antibacterial activity against both the bacterial strains compared to amikacin alone.	[60]
Cefotaxime	Cephalosporin	All possess spherical shape and are monodispersed. Average size of pure AuNPs and Cefotaxime conjugated AuNPs were reported as 6.87 ± 2.43 and 17.55 ± 2.95 nm, respectively.	48 h and 40 °C 542 nm	*E. coli*, *K. pneumoniae*	1.009 μg/mL for *E. coli* and 2.018 μg/mL for *K. pneumoniae*	Conjugation of cefotaxime introduced antibacterial activity to the AuNPs, as pure AuNPs not show any antibacterial properties.	[61]
Vancomycin	Glycopeptide	Spherical shape and monodispersed with an average size 24 nm.	48 h and 40 °C 524 nm	*E. coli*, *K. oxytoca*, *P. aeruginosa*, *S. aureus*	93.44 μg/mL for *E. coli*, 70.84 μg/mL for *K. oxytoca*, 60.65 μg/mL for *P. aeruginosa*, 30.63 μg/mL for *S. aureus*	Vancomycin-coated AuNPs enhanced the antibacterial activity of the drug by 1.6, 1.4, 1.6 and 1.8-fold against *K. oxytoca*, *E. coli*, *S. aureus* and *P. aeruginosa* bacteria.	[62]
Cefoxitin	Cephalosporin	Spherical, poly-dispersed and size between 2 and 12 nm.	48 h and 40 °C 518 nm	*E. coli*, *K. pneumoniae*	MIC 50 value for *E. coli* is 1.5 µg/mL and for *K. pneumoniae* 2.5 µg/mL	AuNPs are found to be efficient for delivering the drug to both the bacterial strains and transform the antibiotic from an unresponsive drug to an effective one.	[32]
Doxycycline	Tetracycline	Spherical, average size was 13 ± 1.2 nm.	15 min 540 nm	*S. aureus* *E. coli*, *K. pneumoniae*, *A. baumannii*, *P. aeruginosa*	2 μg/mL	MIC value for doxycycline conjugated AuNPs was reduced by almost 16 times as compared to doxycycline alone.	[11]
6-Amino-penicillanic acid	-	~3 nm	~1 h in ice water bath	*E. coli*, *K. pneumoniae* *P. aeruginosa*, MDR *E. coli* and MDR *K. pneumoniae*	2.5 μg/mL for *E. coli*, 5 μg/mL for *K. pneumoniae* and for the rest of the bacterial strain, it was 1 μg/mL	MIC value of pure 6-aminopenicillanic acid molecule was greater than 250 μg/mL. But for MDR *E. coli* and *K. pneumoniae* bacteria it was decreased to 5 μg/mL when coated in AuNPs.	[63]
Colistin	Polymyxins	-	-	*E. coli*	0.23 ± 0.03 µg/mL	On conjugation with AuNPs the MIC of colistin reduced by 6.8 fold.	[64]
Imipenem and Meropenem	Carbapenems	35–200 nm	20 min 530 nm	*K. pneumoniae*, *P. mirabilis*, *A. baumanii*	2.5 µg/mL for *K. pneumoniae* And for *P. mirabilis* and *A. baumanii*, MIC value is ~1.25 µg/mL	Imipenem-loaded AuNPs demonstrated decreased MIC of imipenem by four times, and meropenem-loaded AuNPs demonstrated a three times decrease in the MIC of meropenem.	[65]

**Table 3:** Synthesis of some heterocyclic compounds-coated AuNPs to combat AMR.

Heterocyclic Compounds	Size of AuNPs	UV Absorption Peak	Tested Bacteria	Antimicrobial Activity	Refs.
4,6-Diamino- 2-pyrimidinethiol	Less than 4 nm	510 nm	*P. gingivalis*	Inhibited the growth of bacteria as well as biofilm formation and up to a concentration of 10^4^ CFU/mL bacterial growth was arrested.	[81]
Acridine derivatives	15–20 nm	525 nm	*B. subtilis* *E. coli*	9-Aminoacridine hydrochloride hydrate- and acridine orange-coated AuNPs showed enhanced antimicrobial activity.	[82]
3-Amino-1,2,4 triazole- 5-thiol, 6-Amino-2- mercaptobenzothiazole, 2-Amino-6-mercaptopurine, 6-Aminopenicillanic acid, 2-Mercaptoimidazole	4–7 nm	-	*S. aureus* *E. coli* MDR *K. pneumoniae*	2-Mercaptoimidazole and 3-amino-1,2,4-triazole-5-thiol-coated AuNPs reduced the viability of MRSA by 4.1 and 3.5 logs, respectively. 2-Mercaptoimidazole-coated AuNPs reduced the viability of biofilm produced by MDR *K. pneumoniae*, MDR *E. coli* and *E. coli* by 1.3, 1 and 1.9 logs, respectively.	[83]
4,6-Diamino-2- pyrimidinethiol	12.90 and 16.57 nm for pure and coated AuNPs	520 nm	*E. coli*	Compared to the antimicrobial drug levofloxacin, conjugated AuNPs showed superior activity in curing bacterial infections without affecting the intestinal microflora.	[84]
4-Dimethyl aminopyridinium propylthioacetate	5–20 nm	520 nm	*E. coli*	Conjugation with AuNPs enhanced the antimicrobial properties of the 4-Dimethyl aminopyridinium propylthioacetate ligand. Further, combination of the heterocyclic compound-coated AuNPs with a pefoxacin drug reduced the MIC value by almost half compared to the pure drug.	[85]
